# A Comparative Analysis of the Endocannabinoid System in the Retina of Mice, Tree Shrews, and Monkeys

**DOI:** 10.1155/2016/3127658

**Published:** 2016-02-08

**Authors:** Joseph Bouskila, Pasha Javadi, Laurent Elkrief, Christian Casanova, Jean-François Bouchard, Maurice Ptito

**Affiliations:** ^1^School of Optometry, University of Montreal, Montreal, QC, Canada H3T 1P1; ^2^Biomedical Sciences, Faculty of Medicine, University of Montreal, Montreal, QC, Canada H3T 1J4; ^3^Faculty of Medicine, University of Montreal, Montreal, QC, Canada H3T 1J4; ^4^BRAINlab and Neuropsychiatry Laboratory, Department of Neuroscience and Pharmacology, University of Copenhagen, 2200 Copenhagen, Denmark

## Abstract

The endocannabinoid (eCB) system is widely expressed in various parts of the central nervous system, including the retina. The localization of the key eCB receptors, particularly CB1R and CB2R, has been recently reported in rodent and primate retinas with striking interspecies differences. Little is known about the distribution of the enzymes involved in the synthesis and degradation of these eCBs. We therefore examined the expression and localization of the main components of the eCB system in the retina of mice, tree shrews, and monkeys. We found that CB1R and FAAH distributions are well-preserved among these species. However, expression of NAPE-PLD is circumscribed to the photoreceptor layer only in monkeys. In contrast, CB2R expression is variable across these species; in mice, CB2R is found in retinal neurons but not in glial cells; in tree shrews, CB2R is expressed in Müller cell processes of the outer retina and in retinal neurons of the inner retina; in monkeys, CB2R is restricted to Müller cells. Finally, the expression patterns of MAGL and DAGL*α* are differently expressed across species. Overall, these results provide evidence that the eCB system is differently expressed in the retina of these mammals and suggest a distinctive role of eCBs in visual processing.

## 1. Introduction

Marijuana contains over 70 cannabinoids that mimic the endogenous ligands called endocannabinoids (eCBs) that cause global psychoactive and physiological effects. The eCB system is mainly composed of the specific G-protein-coupled receptors CB1R and CB2R, the eCBs (anandamide and 2-arachidonoylglycerol), the synthesising enzymes NAPE-PLD (*N*-acyl phosphatidylethanolamine-specific phospholipase D) and DAGL*α* (diacylglycerol lipase alpha), and the degradation enzymes FAAH (fatty acid amide hydrolase) and MAGL (monoacylglycerol lipase). The cannabinoid receptors are found in many mammals and in various classes of vertebrates and invertebrates and in all major subdivisions of bilaterians, urochordates, and cephalochordates but not in the nonchordate invertebrate phyla like insects [1–3]. The cannabinoid receptors may have evolved in the last common ancestor of the bilaterians with a secondary loss in the insects and other clades [1]. The enzymes responsible for the biosynthesis and the degradation of eCBs are present throughout the animal kingdom [4, 5]. For example, in the rat hippocampus, cerebellum, and amygdala, the distribution of the cytosolic enzyme MAGL is complementary to FAAH (presynaptic versus postsynaptic) suggesting different roles for these two eCBs in the central nervous system (CNS) [6]. The eCB system appears widely distributed in the CNS and points to a fundamental modulatory role of eCBs in the control of many central and peripheral biological functions [7]. A number of specific roles have been ascribed to the eCB system in biological functions, such as neuroprotection, neurogenesis, axon guidance, synaptic plasticity, nociception, motor activity, and memory [8–12]. Disturbances of normal eCB activity may therefore be associated with various brain disorders [13–16].

The eCB system is also found in the retina of various species [17] albeit noticeable differences in its anatomical organization. Compared to rodents, the retina of tree shrews is more similar to primates [18]. Mice have a rod-dominated retina that is specialized for scotopic conditions [19] with a low visual resolution [20]. Mouse and tree shrew retinas have no fovea compared to primates. However, tree shrews have a well-developed binocular visual system, with a cone-dominated retina [21].

In the retina, the expression of CB1R is well-preserved in many species including mice, rats, chicks, larval tiger salamanders, goldfish, and rhesus monkeys [22]. CB1R and CB2R are also present in various retinal cell types (cones, bipolar, ganglion, horizontal, and amacrine cells) with however some differences [17, 23–27]. For example, CB2R is expressed throughout the mouse retina [25] but it is present exclusively in the Müller cells of the vervet monkey [24]. In the mouse retina, DAGL*α* and MAGL are widely distributed throughout the IPL, whereas MAGL is present in rod spherules and cone pedicles of the OPL [28]. Both MAGL and DAGL*α* have been found in an overlapping pattern with CB1R and CB2R in the rat retina. In rats, DAGL*α* is expressed from the early stages of development in photoreceptors, horizontal, amacrine, and ganglion cells and MAGL later during development mainly in amacrine and Müller cells [29]. The expression and distribution of the major components of the eCB system, notably the metabolizing enzymes (NAPE-PLD, DAGL*α*, FAAH, and MAGL), in the retina of different mammals have not been studied in depth. It is therefore our aim to analyze the expression of several components of the eCB system and to characterize their distribution pattern in the distinct retinal layers and cell types of three different mammalian species: mice, tree shrews, and monkeys (vervets and macaques).

## 2. Materials and Methods

### 2.1. Biological Material

Eyes from 3 adult mice (C57BL/6; 3-4 months old), 2 adult tree shrews (*Tupaia belangeri*; 3-4 months old), 3 vervet monkeys (*Chlorocebus sabaeus*; 3-4 years of age), and 2 rhesus monkeys (*Macaca mulatta*; 3-4 years of age) were used in this study. The animals were part of ongoing research projects that were approved by the University of Montreal and McGill University Animal Care and Use Committees. For all species, anterior segment of the eye and vitreous were cut away. The eyecups were bathed in 4% paraformaldehyde made in 0.1 M sodium phosphate buffer at pH 7.4 and left overnight in the solution. The retina was dissected free from the eyecup in a phosphate-buffered saline (PBS) medium. It was laid flat so that the vitreous body could be removed by blotting with filter paper and gentle brushing. Samples of the retina were taken at the center and periphery. Each sample was then cryoprotected in 30% sucrose overnight and embedded in Shandon embedding media at −65°C. The blocks were cut in 20 *μ*m sections at −18°C with a Leica CM3050S cryostat and mounted onto gelatinized subbed glass slides, air dried, and stored at −80°C for further processing.

### 2.2. Immunofluorescence

Single, double, and triple labeling of the retina were performed according to previously published methods [23, 24, 30]. Briefly, the sections were postfixed for 5 minutes in 70% ethanol, rinsed 3 × 5 minutes in 0.1 M Tris buffer and pH 7.4/0.03% Triton and blocked for 90 minutes in 10% normal donkey serum (NDS) in 0.1 M Tris buffer/0.5% Triton. Sections were then incubated with primary antibodies prepared in blocking solution overnight at room temperature. The cannabinoid-related antibodies (CB1R, NAPE-PLD, FAAH, CB2R, DAGL*α*, and MAGL) were also used conjointly with a known specific retinal cell-type marker (Table 1). The next day, sections were washed for 10 minutes and 2 × 5 minutes in 0.1 M Tris/0.03% Triton. Then, they were blocked in 10% NDS and 0.1 M Tris/0.5% Triton for 60 minutes and incubated with secondary antibody for one hour (Alexa 488 donkey anti-mouse and biotinylated donkey anti-rabbit followed by the addition of streptavidin-Alexa 647 (1 : 200), all prepared in blocking solution). Sections were counterstained with Sytox Green Nucleic Acid Stain (1 : 50,000; Molecular Probes, Inc., Eugene, OR), washed again in Tris buffer, and coverslipped with Fluoromount-G*™* Mounting Medium (SouthernBiotech, Birmingham, AL).

### 2.3. Antibody Characterization

**Figure 1:**
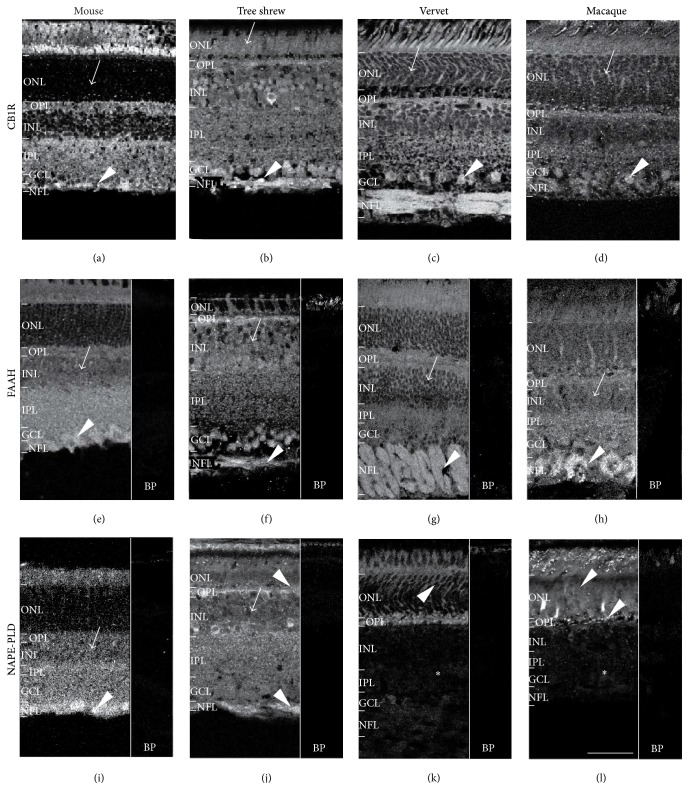
CB1R system immunoreactivity pattern in the retina. Shown are retinal sections immunolabeled for CB1R ((a)–(d)), FAAH ((e)–(h)), and NAPE-PLD ((i)–(l)) in mice, tree shrews, vervet, and macaque monkeys. The control staining, antibody preabsorption with the corresponding blocking peptide (BP), is also shown for FAAH and NAPE-PLD in all species. Arrows point to low to moderate expression of CB1R, FAAH, and NAPE-PLD in the retina of all species and arrowheads to high expression of these proteins. The asterisks indicate undetectable expression of NAPE-PLD in the inner retina of monkeys. ONL: outer nuclear layer; OPL: outer plexiform layer; INL: inner nuclear layer; IPL: inner plexiform layer; GCL: ganglion cell layer; NFL: nerve fiber layer. Scale bar = 75 *μ*m.

**Figure 2:**
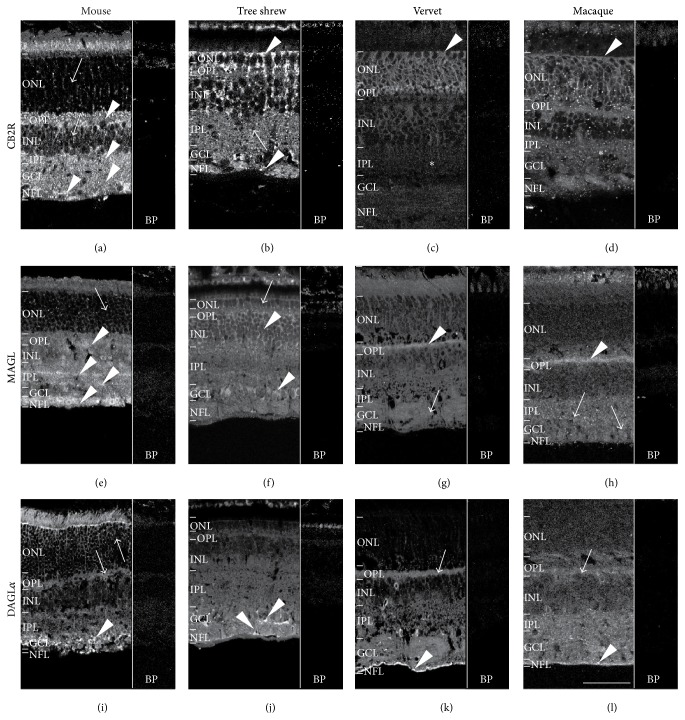
CB2R system immunoreactivity pattern in the retina. Shown are retinal sections immunolabeled for CB2R ((a)–(d)), MAGL ((e)–(h)), and DAGL*α* ((i)–(l)) in mice, tree shrews, vervet, and macaque monkeys. The control staining, antibody preabsorption with the corresponding blocking peptide (BP), is also shown for CB2R, MAGL, and DAGL*α* in all species. Arrows point to low to moderate expression of CB2R, MAGL, and DAGL*α* in the retina all species and arrowheads to their high expression. The asterisks indicate expression of CB2R under the detection level in the inner retina of monkeys. ONL: outer nuclear layer; OPL: outer plexiform layer; INL: inner nuclear layer; IPL: inner plexiform layer; GCL: ganglion cell layer; NFL: nerve fiber layer. Scale bar = 75 *μ*m.

In this study, we were confronted with the problem concerning the specificity of some of the antibodies, especially for the tree shrew. Although knockout animals are the best way to test the specificity of antibodies, this model is available only for mice and not for tree shrews and monkeys. We therefore resorted to the use of conventional alternative methods to circumvent this methodological limitation [23–27]. We have previously published Western blot results for mice and vervet monkeys [23–25]. For tree shrews and macaques, the tissue was not made available to us. Therefore, we resorted to the traditional blocking techniques presented in the paper as BP in Figures 1 and 2. Table 1 summarizes the source and the working dilution of all the primary antibodies. The antibodies used in the present study were characterized and published in previous publications: calbindin [23, 31–35], CB1R [23, 26], CB2R [24, 36], DAGL*α* [26], FAAH [23, 27], GS [23, 37–39], MAGL, NAPE-PLD [40], rhodopsin [30, 41], and PKC*α* [23, 26, 27, 34].

### 2.4. Confocal Microscopy

Immunofluorescence images were taken according to [30]. Using a Leica TCS SP2 confocal laser-scanning microscope (Leica Microsystems, Exton, PA), with a 40x (n.a.: 1.25) or a 100x (n.a: 1.40–0.7) objective, images were obtained sequentially from the green, blue, or far-red channels on optical slices of less than 0.9 *μ*m of thickness. All photomicrograph adjustments, including size, color, brightness, and contrast, were done with Adobe Photoshop (CS5, Adobe Systems, San Jose, CA) and then exported to Adobe InDesign (CS5, Adobe Systems, San Jose, CA), where the final figure layout was completed.

## 3. Results

### 3.1. Single-Label Immunofluorescence

#### 3.1.1. CB1R Is Present Throughout the Retina of All Three Species

A fairly consistent retinal distribution pattern of CB1R across all six retinal layers was observed in mice, tree shrews, vervet, and rhesus monkeys, as illustrated in immunolabeled retinal sections (Figures 1(a)–1(d)). The most significant difference between species is the low expression of CB1R in the ONL of mice when compared to all other species (arrows, Figures 1(a)–1(d)). Furthermore, high expression of CB1R is seen in the GCL and NFL of all species (arrowheads, Figures 1(a)–1(d)).

#### 3.1.2. FAAH Expression Is Found Throughout the Retina of All Three Species

FAAH, like CB1R, is well expressed in all retinal layers and in the photoreceptor layer of all species (Figures 1(e)–1(h)). In all species, there is a moderate protein expression in the INL (arrows, Figures 1(e)–1(h)). Remarkably, there is an important expression of FAAH in the NFL of all species (arrowheads, Figures 1(e)–1(h)).

#### 3.1.3. NAPE-PLD Distribution Is Dissimilar between the Species

In mice, NAPE-PLD is widely distributed in all layers but more intensely in the NFL (arrowhead, Figure 1(i)). In tree shrews, NAPE-PLD is found in all six retinal layers, moderately in the INL (arrow, Figure 1(j)) and prominently in the OPL and NFL (arrowheads, Figure 1(j)). Inversely, in both vervet and macaque monkeys, NAPE-PLD is located in the outer retina, mainly in photoreceptors, ONL and OPL (arrowheads in Figures 1(k) and 1(l)), whilst it is undetectable in the inner retinal layers (asterisks, Figures 1(k) and 1(l)).

#### 3.1.4. CB2R Is Differently Expressed among the Species

Unlike CB1R, the immunolabeling pattern of CB2R is not consistent in the 3 species. In mice, CB2R is moderately detectable in ONL and in INL (arrows Figure 2(a)) but strongly expressed in OPL, IPL, GCL, and NFL (arrowheads, Figure 2(a)). In tree shrews, CB2R is expressed throughout all retinal cell layers with more emphasis (contrary to the mouse) in the external layers (ONL) (upper arrowhead, Figure 2(b)) and NFL (lower arrowhead, Figure 2(b)). In both vervets and macaques, CB2R expression is more abundant in ONL (arrowheads, Figures 2(c) and 2(d)) and is very low in the lower layers (INL, IPL, GCL, and NFL) (asterisks, Figures 2(c) and 2(d)).

#### 3.1.5. Localization of MAGL

In mice, MAGL is expressed in the ONL, OPL, INL, IPL, GCL, and NFL (Figure 2(e)). The most prominent staining is observed in the OPL, in the two laminae of the IPL and in the NFL, as previously described [28] (arrowheads, Figure 2(e)). In tree shrews, MAGL is expressed in all layers and most strongly in the INL and GCL (arrowheads, Figure 2(f)). In vervets and macaques, MAGL is expressed mainly in the OPL (arrowheads, Figures 2(g) and 2(h)). It is also found moderately in the IPL and GCL (arrows, Figures 2(g) and 2(h)).

#### 3.1.6. Expression of the DAGL*α*

In mice, DAGL*α* is weakly expressed in the INL, moderately in OPL and ONL (arrows, Figure 2(i)) but more strongly in the IPL (arrowhead, Figure 2(i)). This result is consistent with that obtained in the mouse retina [28] and in the rat retina [29] that showed expression in the two synaptic layers, the OPL and IPL. DAGL*α* is also highly expressed in the GCL and NFL in the mouse retina (Figure 2(i)). In tree shrews, the DAGL*α* is strongly expressed in the GCL and NFL (arrowheads, Figure 2(j)). In vervets and macaques, DAGL*α* is moderately expressed in the OPL (arrows, Figures 2(k) and 2(l)), whilst there is a high expression in the NFL (arrowheads, Figures 2(k) and 2(l)).

### 3.2. Double-Label Immunofluorescence

To verify the retinal cell-type expression, double immunostaining was carried out with each eCB component and a specific molecular marker for retinal cells.

#### 3.2.1. CB1R and Rod Bipolar Cells

**Figure 3:**
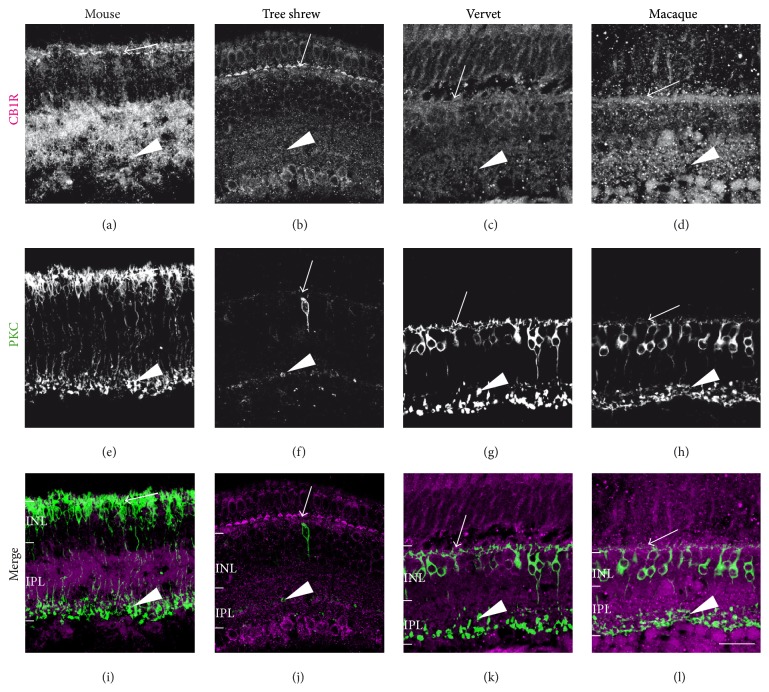
CB1R immunoreactivity in rod bipolar cells. Vertical sections taken from the mouse retina (first column), tree shrew retina (second column), vervet retina (third column), and macaque retina (fourth column). Confocal micrographs of coimmunolabeling for CB1R and the cell-type-specific marker for rod bipolar cells, protein kinase C alpha (PKC*α*). Each protein expression is presented alone in grayscale: CB1R in the first line and PKC*α* in the second line; then the two are presented merged (third line: CB1R in magenta and PKC*α* in green). Arrows point to dendrites ascending into the OPL, where rod spherules are found, and arrowheads point to synaptic terminals in the IPL. INL: inner nuclear layer; IPL: inner plexiform layer. Scale bar = 30 *μ*m.

PKC*α* that labels rod bipolar cells and a subset of amacrine cells is similarly coexpressed with CB1R in the dendrites extending to the OPL (arrows, Figure 3) and synaptic terminals in the IPL in all species (arrowheads, Figure 3). This is in accordance with previous data reported in rats [26] and vervet monkeys [23] by our group.

#### 3.2.2. CB2R and Müller Cells

**Figure 4:**
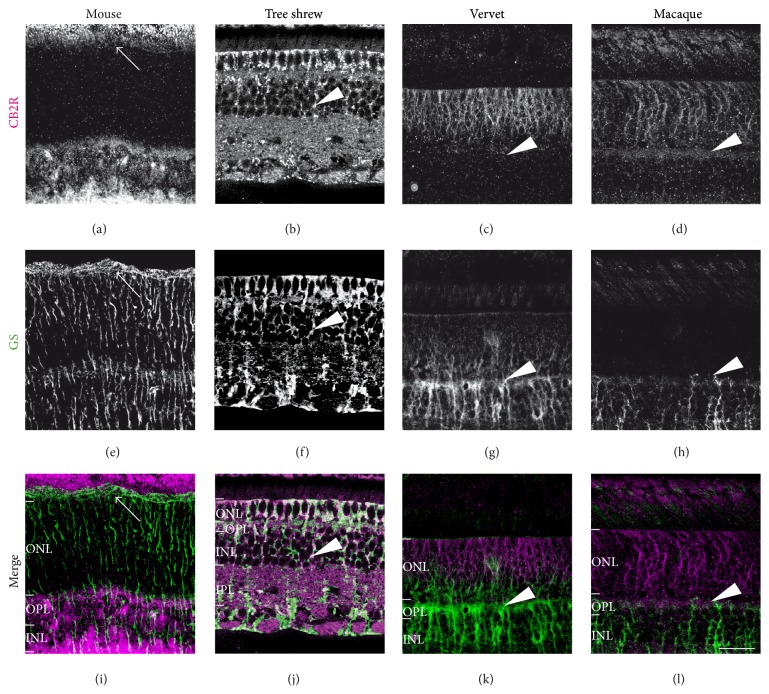
CB2R immunoreactivity in Müller cells. Vertical sections from the mouse retina (first column), tree shrew retina (second column), vervet retina (third column), and macaque retina (fourth column). Confocal micrographs of coimmunolabeling for CB2R and the cell-type-specific marker for glial Müller cells, glutamine synthetase (GS). Each protein immunofluorescent signal is presented alone in grayscale: CB2R in the first line and GS in the second line; then the two are presented merged (third line: CB2R in magenta and GS in green). Arrowheads point to Müller cell processes that all express CB2R, except in mice (arrows). ONL: outer nuclear layer; OPL: outer plexiform layer; INL: inner nuclear layer; IPL: inner plexiform layer. Scale bar = 30 *μ*m.

To label Müller cells, glutamine synthetase (GS) was used. This antibody has proved to be efficient to label Müller cells in the rat [38], mouse [25], and monkey retinas [23, 24, 39]. In mice, CB2R is weakly expressed in the ONL (Figures 4(a), 4(e), and 4(i)) although intense expression was found in the inner layers. CB2R was not found in Müller cells in the mouse retina as previously reported [25]. In tree shrews, CB2R and GS were both expressed in the photoreceptor layer and ONL (arrow, Figures 4(b), 4(f), and 4(j)). Overall, CB2R is colocalized with GS in the outer retina but not in the inner retina (Figure 4(j)). In both vervet and macaque monkeys, double labeling of CB2R and GS shows that CB2R is restricted to Müller cell processes, extending from the internal limiting membrane, with very low staining, to the external limiting membrane, with heavy labeling (arrowheads, Figures 4(c), 4(g), 4(k), 4(d), 4(h), and 4(l)). These results indicate that the expression of CB2R in Müller cells is a feature of tree shrews and monkeys.

#### 3.2.3. NAPE-PLD and Calbindin-Positive Retinal Cells

**Figure 5:**
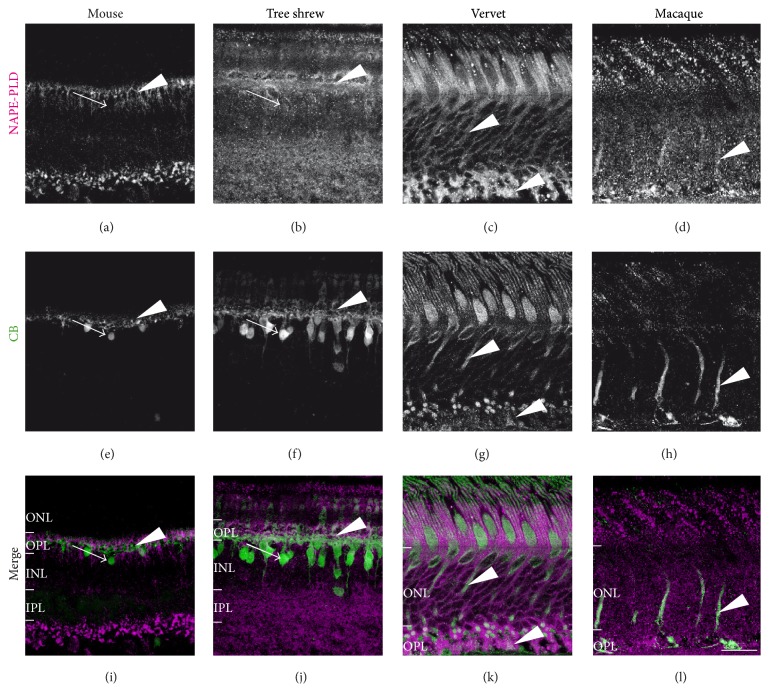
NAPE-PLD immunoreactivity in calbindin-positive retinal cells. Vertical sections from the mouse retina (first column), tree shrew retina (second column), vervet retina (third column), and macaque retina (fourth column). Confocal micrographs of coimmunolabeling for the synthesizing enzyme NAPE-PLD and a cell-type-specific marker for horizontal cells or cones; in mice and tree shrews, calbindin (CB) labels horizontal cells; in monkeys, CB labels cones. Each protein expression is presented alone in grayscale: NAPE-PLD in the first line and the CB in the second line; then the two are presented merged (third line: NAPE-PLD in magenta and the CB in green). Arrowheads point to the processes of CB-positive cells that express the synthetizing enzyme NAPE-PLD and arrows point to CB-positive cells bodies, which do not express NAPE-PLD. ONL: outer nuclear layer; OPL: outer plexiform layer; INL: inner nuclear layer; IPL: inner plexiform layer. Scale bar = 30 *μ*m.

Calbindin (CB) is a marker of cones outside the foveal region, cone bipolar cells, and a subset of horizontal cells in tree shrews and monkeys [23, 32]. On the contrary, in mice, CB is a marker of horizontal cells and is present in OPL with a weak colocalization of NAPE-PLD (Figures 5(a), 5(e), and 5(i)). In both mice and tree shrews CB-positive cell bodies found in the INL do not express NAPE-PLD (arrows, Figures 5(a), 5(e), 5(i), 5(b), 5(f), and 5(j)). In fact, CB is coexpressed with NAPE-PLD in the OPL of tree shrews (arrowheads, Figures 5(b), 5(f), and 5(j)). CB is expressed in the ONL of the monkey retina where NAPE-PLD is abundant (arrowheads, Figures 5(c), 5(g), 5(k), 5(d), 5(h), and 5(l)) and highly coexpressed with NAPE-PLD in the axons of cone photoreceptors (arrowheads, Figures 5(c), 5(g), 5(k), 5(d), 5(h), and 5(l)).

#### 3.2.4. NAPE-PLD and Rods

**Figure 6:**
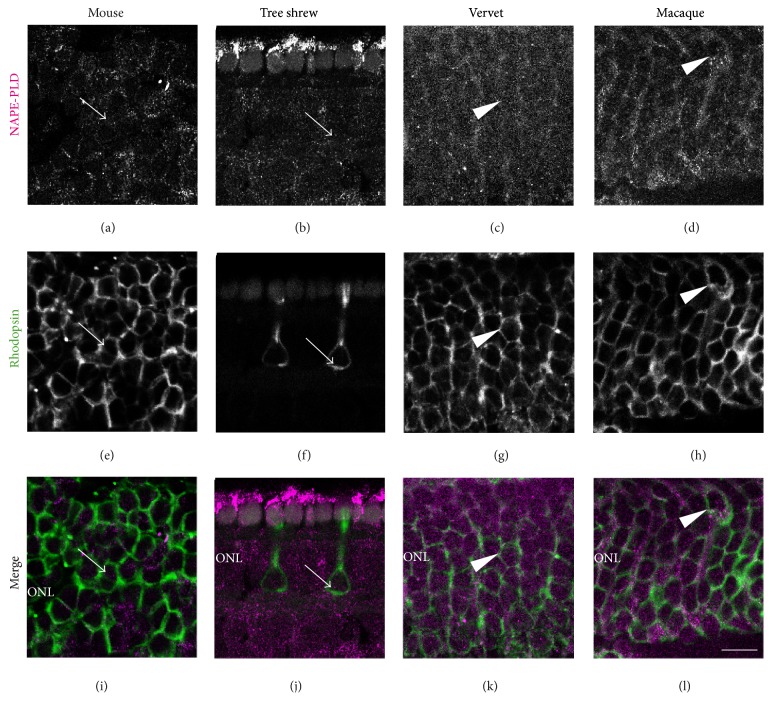
NAPE-PLD immunoreactivity in rod photoreceptors. Vertical sections from the mouse retina (first column), tree shrew retina (second column), vervet retina (third column), and macaque retina (fourth column). Confocal micrographs of coimmunolabeling for the synthesizing enzyme NAPE-PLD and the cell-type-specific marker for rods, rhodopsin. Each immunofluorescent signal is presented alone in grayscale: NAPE-PLD in the first line and rhodopsin in the second line; then the two are presented merged (third line: NAPE-PLD in magenta and rhodopsin in green). Arrowheads point to rhodopsin-positive cell bodies that express NAPE-PLD in vervet and macaque monkeys only, and arrows mark the lack of colocalization. ONL: outer nuclear layer. Scale bar = 30 *μ*m.

The rhodopsin antibody was used to label rods in the retina. In the mouse, NAPE-PLD is not coexpressed with rods (arrows, Figures 6(a), 6(e), and 6(i)). Furthermore, in the cone-dominant retina of the tree shrew with only very few rods, NAPE-PLD is also not colocalized with rods (arrows, Figures 6(b), 6(f), and 6(j)). However, in vervet and macaque monkeys, NAPE-PLD is expressed in rods (arrowheads, Figures 6(c), 6(g), 6(k), 6(d), 6(h), and 6(l)).

## 4. Discussion

**Figure 7:**
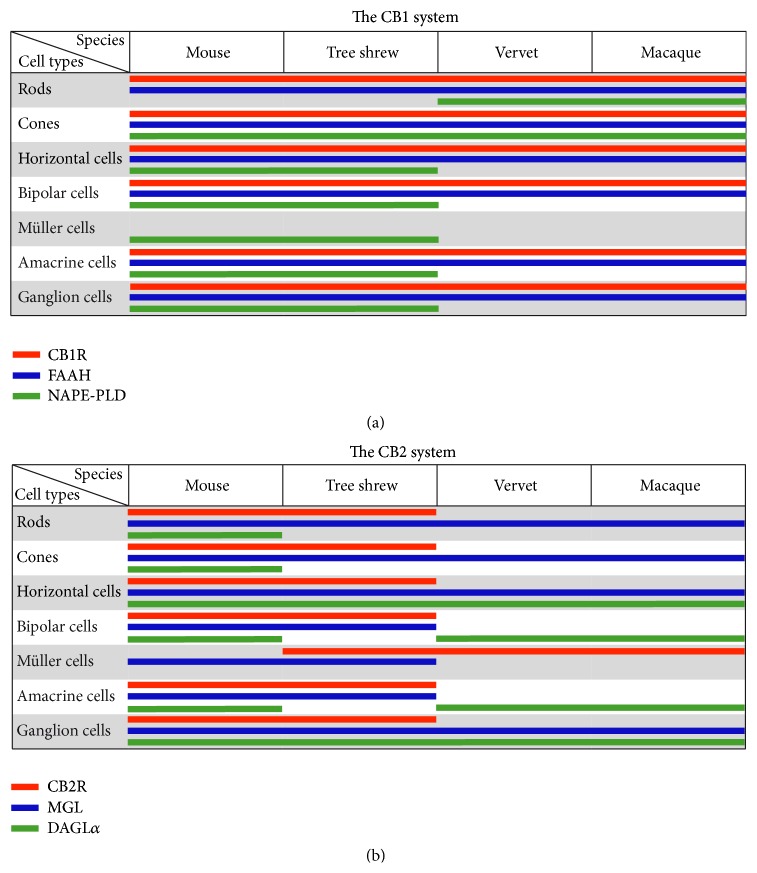
Comparison of the expression patterns of the CB1 system components CB1R, FAAH, and NAPE-PLD (a) and of the CB2 system components CB2R, MAGL, and DAGL*α* (b) in the retina of mice, tree shrews, vervets, and macaques. Our results are complemented by data from previously published work [23, 26, 28, 29].

In this study, we compared the localization of 2 cannabinoid receptors (CB1R and CB2R), 2 endocannabinoid synthesizing enzymes (NAPE-PLD and DAGL*α*), and 2 endocannabinoid degrading enzymes (FAAH and MAGL) in the retina of mice, tree shrews, and monkeys. This is the first study that shows the expression pattern of all the above-mentioned eCB components in the tree shrew retina as well as the localization of the NAPE-PLD, MAGL, and DAGL*α* in the monkey retina (Figure 7). These phylogenetically related species were chosen due to the specialization of their visual systems: from the primitive monocular, rod-dominated visual system in mice with a low visual resolution to the well-developed visual system in monkeys [42] that is similar to humans [43]. Tree shrews are a species with binocular cone-dominated vision that is phylogenetically between mice and monkeys [21, 44].

### 4.1. The Cannabinoid Receptors: Localization versus Function

We recently reported that the distribution of the CB2R in the primate retina [24] is different than the rodent retina [25]. While the CB2R is expressed in the rodent retinal neuronal cells [25], it is only expressed in the primate retinal glia, the Müller cells [24]. This finding prompted us to look into the retinal eCB system expression profiles across species. Interestingly, we show that only some components of the eCB system are preserved across the three animal species studied here while others are strikingly different. Notably, as reported by Elphick in his thought-provoking review [5], CB1R and CB2R are unique to chordates, but the enzymes involved in the biosynthesis and the inactivation of the eCBs like NAPE-PLD and FAAH are found throughout the animal kingdom [4]. These proteins may have therefore evolved as presynaptic or postsynaptic receptors for eCBs. This is fascinating because the expression and localization of CB1R and FAAH are similar in mice, tree shrews, and primates, while it is not the case for CB2R, NAPE-PLD, MAGL, and DAGL*α* (Figure 7).

There are many controversies on the neuronal and/or peripheral expression of CB2R. Our results show that the expression pattern of the CB2R differs from the mouse to the monkey. Similar to CB1R, CB2R shows a general expression in the neuroretina: photoreceptors, horizontal cells, amacrine cells, and cells localized in the GCL of rodents [25, 45]. In the mouse, CB2R, expressed in the photoreceptor layer, was mostly found in cones and some rods [25]. Similar to its position in the phylogeny tree, the tree shrew has an in-between position showing expressions in all layers, as in rodents, and in Müller cells, as in primates (Figure 2(b)). In agreement with the CB2R glial expression in the CNS, the primate retina expresses CB2R mainly in Müller cells, with a higher polarization towards the outer retina [24]. The Müller cells, with their unique anatomy, span the entire thickness of the retina and contact with the majority of the retinal neurons [46]. This complementary expression pattern of CB1R and CB2R in the primate retina reveals thus a reciprocal relationship between retinal neurons and glia regarding their function via the eCB system. The ubiquitous CB1R system may play a more general role in the light transduction in all three species, as previously suggested [17].

### 4.2. Significance of the Distribution Pattern of Enzymes and Cannabinoid Receptors

The expression pattern of CB1R and FAAH has been reported in the CNS as complementary, overlapping, or unrelated distributions [17, 47]. Here, we report an overlapping distribution; CB1R expressing neurons also express FAAH. In this case, the degrading enzyme may remotely influence the CB1R [47]. During development of the mouse retina, CB1R and FAAH expression patterns are present in the deepest neuroblast layers at birth and spread out throughout the retina in adulthood [26, 27]. In our three species, the FAAH expression overlaps the CB1R distribution pattern not only in the photoreceptor layers but also in the ganglion cells (Figures 1(a)–1(d) and 1(i)–1(l)). This suggests that cannabinoids act not only on photoreceptors [17] but also directly on ganglion cell. This expression pattern has been reported not only in the retina of the vervet monkey [23] but also in the optic nerve, the dorsal lateral geniculate nucleus [40], and the visual cortex of monkeys [48]. While NAPE-PLD and FAAH are overlapping in different layers of the mouse and tree shrew retinas, they are complementarily expressed in the monkey retina. This unique complementary spatial relationship between NAPE-PLD (exclusively in the photoreceptor layer) and FAAH (in the inner retina) might ensure optimal retinal function in highly developed retinas. However, further experiments are needed to test this hypothesis.

Anandamide (an endogenous agonist of the CB1R) and other* N*-acylethanolamines (NAEs) are biosynthesized from phospholipids of the cell membrane assisted by NAPE-PLD hydrolysis. In this study, we report a variation in the expression of this membrane associated synthesis enzyme, NAPE-PLD, despite its well-preserved sequence from rodents to humans [49]. In the mouse, NAPE-PLD follows the same pattern of expression as CB1R and FAAH, except that it is not found in rods. Moreover, unlike the mouse but like the primate, the tree shrew has a high expression of NAPE-PLD in ONL and OPL. We show here for the first time that NAPE-PLD expression in monkeys is exclusively restricted to the photoreceptor layer. Unlike CB1R, NAPE-PLD is ubiquitously expressed in the rat brain with the highest level in the thalamus [50]. Besides its role in the eCB biosynthesis, many other physiological roles have been linked to NAPE-PLD such as anti-inflammatory effect [51], anorexic effect [52], and proapoptotic effect [53]. Moreover, the NAE products in axons suggest a role in the regulation of postsynaptic neuron activity as anterograde synaptic signaling molecules [54]. This pattern of expression also suggests another direct role of NAEs in primate phototransduction.

Given that the lipophilic eCBs are released and degraded close to their action site, it would be reasonable to assume that the DAGL*α* and MAGL expressions are in the vicinity of CB2R. In the mouse retina, the DAGL*α* and MAGL expressions are often near or in the same cell types as CB1R and CB2R. CB1R is present in cones, horizontal, bipolar, amacrine, and ganglion cells in the rat retina [26, 27]. CB2R is present in cone and rod photoreceptors, horizontal, bipolar, amacrine, and ganglion cells in the adult mouse retina [25]. This distribution pattern may suggest that, in the mouse retina, eCBs such as 2-arachidonoyl glycerol are faithfully expressed adjacent to the cannabinoid receptors and could be involved in their retinal function [25]. But the primates and the tree shrews have followed a complementary distribution pattern, and may have adopted a more complex and specific strategy to regulate their visual activity via the eCB system. The eCB expression pattern in the mouse rod-dominated retina with monocular vision, the tree shrew cone-dominated retina with binocular vision, and the monkey duplex retina with binocular vision proposed that the retinal eCB system plays a fundamental role in the mammal visual processing.

## Figures and Tables

**Table 1:** List of antibodies used in this study.

Antibody^1^	Immunogen	Source^2^	Working dilution
CB	Purified bovine kidney calbindin-D28K	Sigma-Aldrich, St. Louis, MO; C9848, mouse monoclonal, clone CB0955	1 : 250
CB1R	Fusion protein containing aa 1–77 of rat CB1R	Calbiochem, Gibbstown, NJ; 209550, rabbit polyclonal	1 : 150
CB2R	Synthetic peptide corresponding to aa 20–33 of human CB2R	Cayman Chemical, Ann Arbour, MI; 101550, rabbit polyclonal	1 : 150
DAGL*α*	Peptide with sequence CPAKQDELVISAR, from the C Terminus of the protein sequence	Novus, Littleton, CO; NBP2-31856, rabbit polyclonal	1 : 100
FAAH	Synthetic peptide aa 561–579 of rat FAAH	Cayman Chemical, Ann Arbour, MI; 101600, rabbit polyclonal	1 : 150
GS	Full protein purified from sheep brain	Chemicon, Temecula, CA; MAB302, mouse monoclonal, clone GS-6	1 : 500
MAGL	Human MAGL aa 1–14	Cayman Chemical, Ann Arbour, MI; 100035, rabbit polyclonal	1 : 150
NAPE-PLD	Purified protein corresponding to aa 159–172 NAPE-PLD human	Cayman Chemical, Ann Arbour, MI; 10305, rabbit polyclonal	1 : 200
Rhodopsin	Bovine rhodopsin	Abcam, Toronto, ON; ab98887, mouse monoclonal, clone Rho 4D2	1 : 500
PKC*α*	Peptide mapping the aa 296–317 of human PKC*α*	Santa Cruz Biotechnology, Santa Cruz, CA; sc-8393, mouse monoclonal, clone H-7	1 : 500

^1^CB: calbindin; CB1R: cannabinoid receptor type 1; CB2R: cannabinoid receptor type 2; DAGL*α*: diacylglycerol lipase alpha; FAAH: fatty acid amide hydrolase; GS: glutamine synthetase; MAGL: monoacylglycerol lipase; NAPE-PLD: *N*-acyl phosphatidylethanolamine-specific phospholipase D; PKC*α*: protein kinase C *α* (alpha isoform); aa: amino acids.

^2^The source column indicates the commercial company, catalog reference, and origin. The clone designation is given for monoclonal antibodies.
